# Feeding *Drosophila* gut microbiomes from young and old flies modifies the microbiome

**DOI:** 10.1038/s41598-024-58500-1

**Published:** 2024-04-02

**Authors:** Jonas Bruhn Wesseltoft, Christian Dupont Danielsen, Andreas Mølgaard Andersen, Nadieh de Jonge, Anders Olsen, Palle Duun Rohde, Torsten Nygaard Kristensen

**Affiliations:** 1 Department of Chemistry and Bioscience, Aalborg University, Aalborg, Denmark; 2 Department of Health Science and Technology, Aalborg University, Aalborg, Denmark

**Keywords:** Drosophila, DNA, DNA sequencing, Next-generation sequencing, Microbiome, Ageing

## Abstract

It is becoming increasingly evident that the myriad of microbes in the gut, within cells and attached to body parts (or roots of plants), play crucial roles for the host. Although this has been known for decades, recent developments in molecular biology allow for expanded insight into the abundance and function of these microbes. Here we used the vinegar fly, *Drosophila melanogaster*, to investigate fitness measures across the lifetime of flies fed a suspension of gut microbes harvested from young or old flies, respectively. Our hypothesis was that flies constitutively enriched with a ‘Young microbiome’ would live longer and be more agile at old age (i.e. have increased healthspan) compared to flies enriched with an ‘Old microbiome’. Three major take home messages came out of our study: (1) the gut microbiomes of young and old flies differ markedly; (2) feeding flies with Young and Old microbiomes altered the microbiome of recipient flies and (3) the two different microbial diets did not have any effect on locomotor activity nor lifespan of the recipient flies, contradicting our working hypothesis. Combined, these results provide novel insight into the interplay between hosts and their microbiomes and clearly highlight that the phenotypic effects of gut transplants and probiotics can be complex and unpredictable.

## Introduction

Most animal and plant species are holobionts meaning that, in addition to their own genomic makeup, they carry the vast genomic gene complement of all their associated microbiomes^1^. Thus, the host genomics and its associated microbial diversity constitutes a complex community which collectively play a crucial role in several physiological processes important for the host including, nutrient acquisition^2^, development^3^ and immune response^4^. The important interactions between hosts and microbiomes are a result of co-evolution and they are often of a mutualistic character^5^.

Since the Human Microbiome Project was published^6^, an enormous effort has been put into describing and characterizing the human microbiome which has led to a substantial increase in our understanding of what shapes the human microbiome diversity^7,8^. It has been shown that the human microbiome is not only highly diverse and dynamic but also that alterations in the microbial composition can result in drastic phenotypic effects for the host^9^. For example, specific human gut microbial compositions have been associated with a range of non-communicable diseases including, irritable bowel syndrome^10^, diabetes^11^, the susceptibility to infections^12^ and even various neurological disorders^13^.

Knowledge obtained on the significance of the microbiome–host interactions is of key interest within many biological sciences such as evolutionary and conservation biology^14,15^. For example, certain microorganisms play an important role in rapid adaptation of *Caenorhabditis elegans* to environmental perturbations^16^. These adaptations can occur in the form of the production of necessary nutrients^17^ or beneficial compounds like short chain fatty acids (SCFA)^18^, increased resistance to temperature fluctuations^19^ or toxic compounds^20^, all functions provided by microbial partners. These ‘services’ provided by microbes can enable the host to thrive under environmental conditions that they would not be able to tolerate otherwise. The dynamic nature of the microbial composition and their rapid evolution, suggests a role of host-microbe interactions in host resilience to fast environmental changes and stress^21^. Thus, the ability of hosts to cope with increasing and more fluctuating temperatures induced by climate change, could be limited by the thermal tolerance of their microbial partners^22^. Additionally, anthropogenic factors such as habitat fragmentation leading to population bottlenecks can result in a decrease in host microbiome diversity^23,24^ and loss of functional members, like SCFA producers, possibly resulting in a decrease in fitness of the host organism^25^.

The many important roles that the microbiome plays in disease and general performance (fitness) of individuals and populations has been a driver for the increasing interest and research on this topic over the last decades. This work includes attempts to alter/manipulate the microbiomes of especially humans but also other animals and plants to provide the host with certain desired traits^26^. In humans, one goal with this type of research is to expand the period of life in which an individual is in good health, commonly referred to as healthspan. This has become an increasingly useful therapeutic tool executed in the form of fecal microbiota transplants which are being used to treat a variety of diseases and conditions including *Clostridium difficile* infections^27^ and irritable bowel syndrome^28,29^.

Probiotics, the supplementation of beneficial commensal bacteria to existing microbiomes, is of interest in both industrial and scientific communities. For example, probiotics has been proposed as an alternative or supplement to antibiotics treatments for livestock and pets^30^ as well to increase robustness of wildlife species which have become endangered due to anthropogenic factors^31^. Likewise, manipulation of specific host bacteria has been proposed as a way to control pest insect species of importance in agriculture and human diseases^32^. The idea here is that introducing self-spreading endosymbiont bacteria to pest species reduces their fitness and this can therefore be an alternative way to control them.

Because of the important roles of microbiomes in relation to healthspan of humans and many animals, it is also being investigated from the angle of ‘healthy aging’, which grows increasingly relevant with a human population rapidly increasing in average lifespan. Studies suggest that the microbiomes of old healthy humans are distinguishable from that of elderly suffering from frailty or other co-morbidities, namely in that they are significantly more diverse and contain community-associated microbes^33^.

The composition, and thus, functional potential of the microbiome is maintained by a carefully tuned immune system that is required to respond to the emergence of pathogenic microorganisms while fostering a mutualistic environment for the commensal bacteria in the gastrointestinal tract^34^. For this reason, it is not surprising that as an individual ages and experiences reduced immunocompetence, like chronic inflammation, this will have drastic effects on the microbiome^35^. For example, an age-dependent chronic activation of the FOXO pathway in *Drosophila melanogaster* results in commensal dysbiosis^36^, and most of the conserved age-related changes in gene expression are dependent on the presence of a certain bacterial cohort^37^. Targeting this age dependent microbial dysbiosis has been suggested as a method for increasing the period of which the host is able to maintain a functional relationship between host and microbiome, and thus increasing both the lifespan and healthspan of the host^35,36,38,39^.

Probing the complex interactions between microbiomes, host response, aging and environment in humans and wild living metazoans can prove wildly complex since it is hard to control for the many factors that can influence the delicate interactions^40^. For this reason, much of the research that is being performed in this field makes use of model organisms like *C. elegans*^18,41,42^ or *D. melanogaster*^43–46^. Here we used *D. melanogaster* to investigate whether enriching the gut microbiome of flies by feeding them microbiomes from flies of different ages, altered the microbial composition and whether traits related to healthspan and lifespan were affected by the microbial enrichment. This question has never been directly assessed before and results therefore have the potential to provide know-how that can later be verified in mammalian model species and eventually humans. *D. melanogaster* is a well-suited model species for this type of experiment because we can easily manipulate and control diets, follow healthspan throughout life, and within a short timeframe (few months) provide data that would require decades of work if studies were to be performed on humans. Our hypothesis was that, by actively shaping the development of the microbiome towards that of younger individuals, it was possible to increase fitness traits by postponing age-related gut dysbiosis to later in life. Multiple experimental evidence supports this hypothesis. First there is evidence that diverse microbiomes constitute a host fitness benefit in *D. melanogaster*^23^ and that gut microbiomes of healthy old people are more diverse than those of unhealthy old people^33^. Secondly in killifish^39^ where transplantation of ‘young’ microbiomes into middle aged fish treated with an antibiotic cocktail resulted in an increase in lifespan and fitness. However, such harsh antibiotic treatments can be detrimental for the host^47,48^ or not a suitable method of intervention^49,50^ and for this reason it is of great interest to test whether it is possible to shape a naturally existing microbiome by supplementing with differently aged microbiomes, in order to investigate phenotypic effects.

## Materials and methods

### Fly husbandry and microbial enrichment

#### *D. melanogaster* population and preparation of microbial suspensions

The mass-bred population of *D. melanogaster* used in this study was founded by approximately 600 inseminated females caught in Odder (55° 56042.4600 N, 10° 12045.3100 E), Denmark, in October 2010. Flies were maintained at 23 °C and 50% RH at a 12:12 h light/dark cycle. Prior to the experiments, flies were reared on standard Leeds medium composed of dry yeast (60 g L^−1^), sucrose (40 g L^−1^), oatmeal (30 g L^−1^), agar (16 g L^−1^), Nipagen (12 mL L^−1^) (Nipagen, Sigma-Aldrich) and acetic acid (1.2 mL L^−1^). To produce the flies used for producing microbial suspensions, 600 flies (mixed sex) were distributed with 100 individuals in each of six bottles from the mass-bred population and kept at 20 °C for 24 h. Eggs were collected and distributed with 40 eggs in each of 50 vials with standard Leeds medium. At emergence, females and males were anesthetized with CO_2_ and males were transferred to new vials (females were discarded) containing 20 individuals per vial. Flies were kept at 20 °C and 50% RH at a 12:12 h light/dark cycle.

20 newly (0–24 h) eclosed *D. melanogaster* males were isolated and reared in each of 50 vials (94 × 25 mm) containing 3 mL Leeds medium. Flies were transferred to vials with fresh Leeds medium every third day. When flies were either 3 or 77 days old (last surviving flies), we anesthetised them and dissected intestinal tracts. While kept cold on ice intestinal tracts were dissected and microbial suspensions were prepared by adding the ground (using pestles) intestinal tract including gut contents of 11 flies to 2200 µL 5% autoclaved sucrose solution for each tube following a modified protocol from Tauc et al.^51^. In total 22 Eppendorf tubes with suspensions made up of either young (3 days old) or old (77 days old) intestinal tracts were made. Additionally, a control solution was created in Eppendorf tubes that only contained 5% autoclaved sucrose. All tubes were stored at − 80 °C until use. The three suspension types were denoted Young, Old and Control.

#### Microbial enrichment

**Figure 1:**
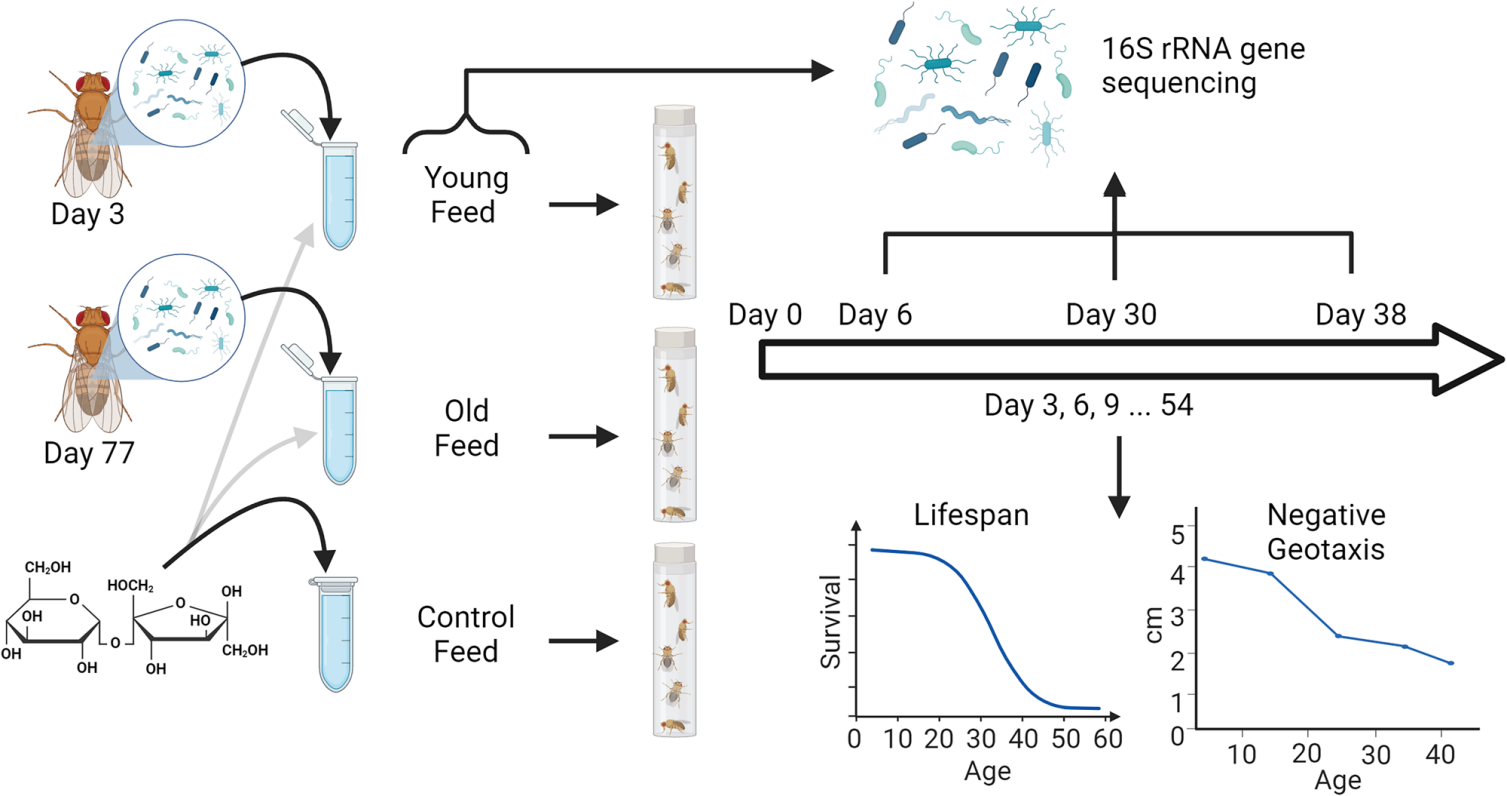
Flow Chart illustrating the experimental steps. Intestinal tracts from three-day old flies (Young) and 77 days old flies (Old) were dissected and added to a 5% sucrose solution. The two different microbial solutions, or a sucrose Control solution were fed to flies. Flies were scored every three days for survival and negative geotaxis (provides a measure of behavioral activity). At days 3, 30 and 38, flies were frozen and subsequently used for 16S rRNA gene sequencing alongside the feed solutions. Created with BioRender.com**.**

We produced flies to be used in an experiment where flies were fed the three types of feed suspensions in the following way: 600 flies (mixed sex) were distributed across each of 4 bottles from the same mass-bred population as described above and kept at 23 °C for 24 h. Eggs were collected and distributed with 40 eggs in each of 50 vials with standard Leeds medium. At emergence, females and males were anesthetized with CO_2_ and males (females were discarded) were transferred to new empty vials each with five newly eclosed (0-24 h) male flies. Flies were kept at 23 °C with ca. 99% RH and at a 12:12 h light/dark cycle. 26 vials were prepared for each of the Young enrichment feed, Old enrichment feed and the Control feed, adding to 130 flies for each treatment. 16 vials of each treatment were used for longevity assay while 10 were used for the microbiome assay. To each of these vials, an Eppendorf tube lid (8 mm diameter) was glued to the side of the vials (see Fig. 1—flow chart) functioning as a feed container which the flies could access. 80 µL Old, Young or Control suspensions were added to these lids every three days (after cleaning the lids and vials with ethanol). Suspensions were taken from the freezer and thawed right before adding new solutions to the lids.

### Longevity assay

Every three days, starting at day 0, all flies were transferred to new vials and the number of dead individuals were recorded. This process was continued until all flies in each of 48 vials were dead. Flies that escaped or died during transfer were censored from the data.

### RING assay

For this purpose, we utilized a modified version of the high-throughput Rapid Iterative Negative Geotaxis (RING) assay^52,53^. In this assay digital photography was used to document negative geotaxis behaviour of flies. To assess negative geotaxis, the five (progressively fewer as the experiment progresses and flies start to die or are harvested for other purposes) flies in each of the 78 vials (26 from each of the 3 treatments) were transferred to empty vials (94 × 25 mm). Five minutes after the flies had been transferred to the vials all vials were forcefully knocked down three times in rapid succession to initiate the geotaxis response. A photo of the vertical position of the flies was captured exactly 4 s after eliciting the behaviour. This was performed a total of five times with 30 s pause in between. Images of the flies’ positions were captured with an acA1300–60 gm GigE camera (Basler). The mean height of the flies within each vial was measured using ImageJ software (version 1.48). All RING experiments were conducted in a climate-controlled room at 23 °C, 50% RH and at constant light. Negative geotaxis behaviour was assessed between 13:00 and 17:00 a.m. on each test day (the locomotor activity in *Drosophila* exhibits a distinct circadian rhythm^54^). An increased negative geotaxis behaviour, i.e. the flies crawled higher, was interpreted as beneficial, i.e. more agile and fit flies^55^. After completion of the RING assay the flies were transferred back to vials with Old, Young and Control solutions as feed sources. The RING assay was performed every three days, starting at day 0.

### Sample collection and DNA extraction for microbiome analysis

At ages 6, 30 and 38 days, eight surviving flies were collected from each treatment group (Young, Old and Control feed), and split into two pools of four individuals. Flies were collected randomly from available vials when possible, ensuring that flies were collected from all vials. The last flies for the microbiome analysis were collected at day 38. Originally, we had aimed for a more even distribution, collecting the oldest flies around day 54. However, flies died faster than expected and therefore we harvested the last flies at 38 days of age. All samples were stored at − 80 °C until DNA extraction. The total genomic DNA was extracted from each pool of flies and from triplicate samples from each suspension type using the DNeasy Blood and Tissue kit (QIAGEN) according to the manufacturer’s instruction for DNA extraction of insects. Agilent TapeStation 2200 was used alongside Genomic DNA ScreenTape (Agilent Technologies) to assess the quality of the DNA extracts, and Qubit BR dsDNA kit (Thermo Fisher Scientific) and a Tecan Infinite F200 PRO (Tecan Life Sciences) was used for estimating DNA concentrations.

### 16S rRNA gene amplicon sequencing

The hypervariable V1-3 region of bacterial 16S rRNA gene was amplified by PCR, using the V1-3 primer set 27F/534R from the Human Microbiome Project^6^, fused with Nanopore PCR barcoding overhangs. PCR was performed in duplicates, in a total reaction volume of 25 µL (1X PCRBIO Ultramix (PCR Biosystems), 400 nM of each primer and 4 µL template DNA). Thermocycler settings were as follows: Initial Denaturation at 95 °C for, 2 min, followed by 35 cycles of: 95 °C for 15 s., 56 °C for 15 s. and 72 °C for 60 s, followed by a final extension at 72 °C for 5 min. Duplicate PCR reactions were subsequently pooled, and the generated amplicons were purified using CleanNGS magnetic beads using a 0.8 bead to sample ratio (CleanNA). All samples were barcoded with the PCR Barcoding Expansion 96 kit EXP-PBC096 (Oxford Nanopore Technologies) and subsequently prepared for sequencing according to manufacturer specifications (PBAC96_9069_v109_revQ_14Aug2019) using the NEBNext Companion Module E7180 (New England Biolabs) and Ligation Sequencing Kit SQK-LSK110 (Oxford Nanopore Technologies). Sequencing libraries were loaded onto a FLO-MIN106D R9 Nanopore flow cell and sequenced using a GridION device for 7 h. Between steps, DNA quality and quantity were verified using Agilent TapeStation 2200 and D1000 DNA ScreenTape (Agilent Technologies) Qubit BR dsDNA kit (Thermo Fisher Scientific) and Tecan Infinite F200 PRO (Tecan Life Sciences).

### Sequencing data treatment

Raw sequences were base called using Guppy v6.5.7 with the R 9.4.1 high accuracy model using MinKNow version 23.11.3 (https://community.nanoporetech.com/downloads). Demultiplexing, adapter removal and initial quality control was performed using Porechop v0.2.4, using the options -require_two_barcodes, -discard_middle and -barcode_threshold 85 for stringent quality filtering. The resulting data was quality checked using Nanoplot 1.24.0. This was followed by a filtering step by NanoFilt 2.6.0 where only sequences of lengths between 150 and 600 bp with a quality score above 10 were retained. The primer sequences were removed with cutadapt 3.4. Filtered reads were aligned and polished once using minimap 2.24 and Racon 1.5.0 respectively. Polished reads were clustered into Operational Taxonomic Units (OTU’s) using VSEARCH 2.13.4 at a 97% sequence similarity and taxonomy was assigned against the SILVA v138 database. The resulting OTU data was manually curated to retain only OTUs with a minimum read count of 3. Additionally, the genera *Legionella* and *Ralstonia* were manually filtered out as these are common contaminants from DNA extraction kits^56^ and were only observed with a relative abundance above 0.5 in the control feed solution samples.

### Data analysis and statistics

All presented data were analysed using R version 4.3.1 and RStudio version 2023.06.1-524^57^. Alpha and beta diversity analyses, and heatmaps were generated using the Ampvis2 package^58^. Alpha diversity was quantified using Shannon and Simpson diversity indices along with the observed diversity (OTU counts). Differences between groups in these measures were investigated using a pairwise Wilcoxon ranked sum test with Benjamini–Hochberg multiple test correction. Beta diversity was presented with a correspondence analysis (CA) on Hellinger transformed Bray–Curtis distances and differences were tested using ANOSIM with 999 permutations. Log rank test was performed to determine feed solution effects on lifespan while a Two-way ANOVA was performed for the RING assay testing for effects of feed solution, age, and their interaction.

## Results

16S rRNA gene amplicon sequencing of the V1–V3 region revealed a total count of 631,117 reads across all 27 samples (Table 1) with an average of 23,374 ± 1902 reads per sample (mean ± SE). A beginning horizontal asymptote tendency was observed in the rarefaction curve (Fig. S1), suggesting a sequencing depth sufficient for the relatively simple microbiomes of *D. melanogaster*.

**Table 1:** Alpha diversity index values of the fly and feed suspension microbiomes (mean ± SE).

	Observed OTU diversity (S)	Shannon diversity index (H)	Simpson’s diversity index (D)
Feed suspension—young (n = 3)	464 ± 49	2.46 ± 0.22	0.68 ± 0.05
Feed suspension—old (n = 3)	530 ± 14	3.48 ± 0.23	0.91 ± 0.02
Feed suspension—control (n = 3)	357 ± 51	4.32 ± 0.16	0.96 ± 0.00
Day 6			
Young (n = 2)	480 ± 70	2.76 ± 0.40	0.79 ± 0.10
Old (n = 2)	537 ± 36	2.97 ± 0.01	0.84 ± 0.01
Control (n = 2)	586 ± 50	3.19 ± 0.13	0.87 ± 0.00
Day 30			
Young (n = 2)	690 ± 114	3.50 ± 0.06	0.92 ± 0.00
Old (n = 2)	719 ± 95	3.58 ± 0.12	0.91 ± 0.00
Control (n = 2)	610 ± 31	3.50 ± 0.03	0.91 ± 0.00
Day 38			
Young (n = 2)	826 ± 43	3.89 ± 0.20	0.93 ± 0.02
Old (n = 2)	793 ± 27	3.72 ± 0.07	0.94 ± 0.00
Control (n = 2)	808 ± 10	3.74 ± 0.10	0.93 ± 0.00

Across the 27 samples, a total of 2527 unique OTU’s were recorded and an average of 598 ± 31 OUT’s (mean ± SE) were observed per sample.

### Microbial diversity increases with age across enrichment treatments

In order to investigate the effects of enriching the microbiomes of flies fed with microbial suspensions from juvenile or ageing flies, populations of male *D. melanogaster* were reared on microbial feed suspensions prepared from the gut contents from either young flies, old flies or a sucrose control. From the subsequent microbiome analysis we obtained alpha diversity indices (Table 1).

A significant increase in microbial diversity of both richness and evenness were observed across all fly samples with increasing age of the flies (*p* ≤ 0.05). No significant differences were found in microbial diversity of flies exposed to the different feed treatments, except for a small trend in the 6 days old flies fed with the Young microbial solution, in which diversity was lower and more variable compared to flies fed both the Control and Old feed.

The alpha diversity indices of the microbial feed suspensions showed a marked increase in diversity with increasing age of the flies used to produce the suspensions, as the suspensions from the 77 days old flies had both a higher OTU count as well as a higher evenness as compared to the suspension from the younger flies.

To better understand the differences and similarities between the different feeds and the aging microbiotas, an ordination analysis was performed for all samples (Fig. S2), which showed marked differences between the control feed and the other samples. To reduce noise, we therefore removed the control feed samples from subsequent analyses.

**Figure 2:**
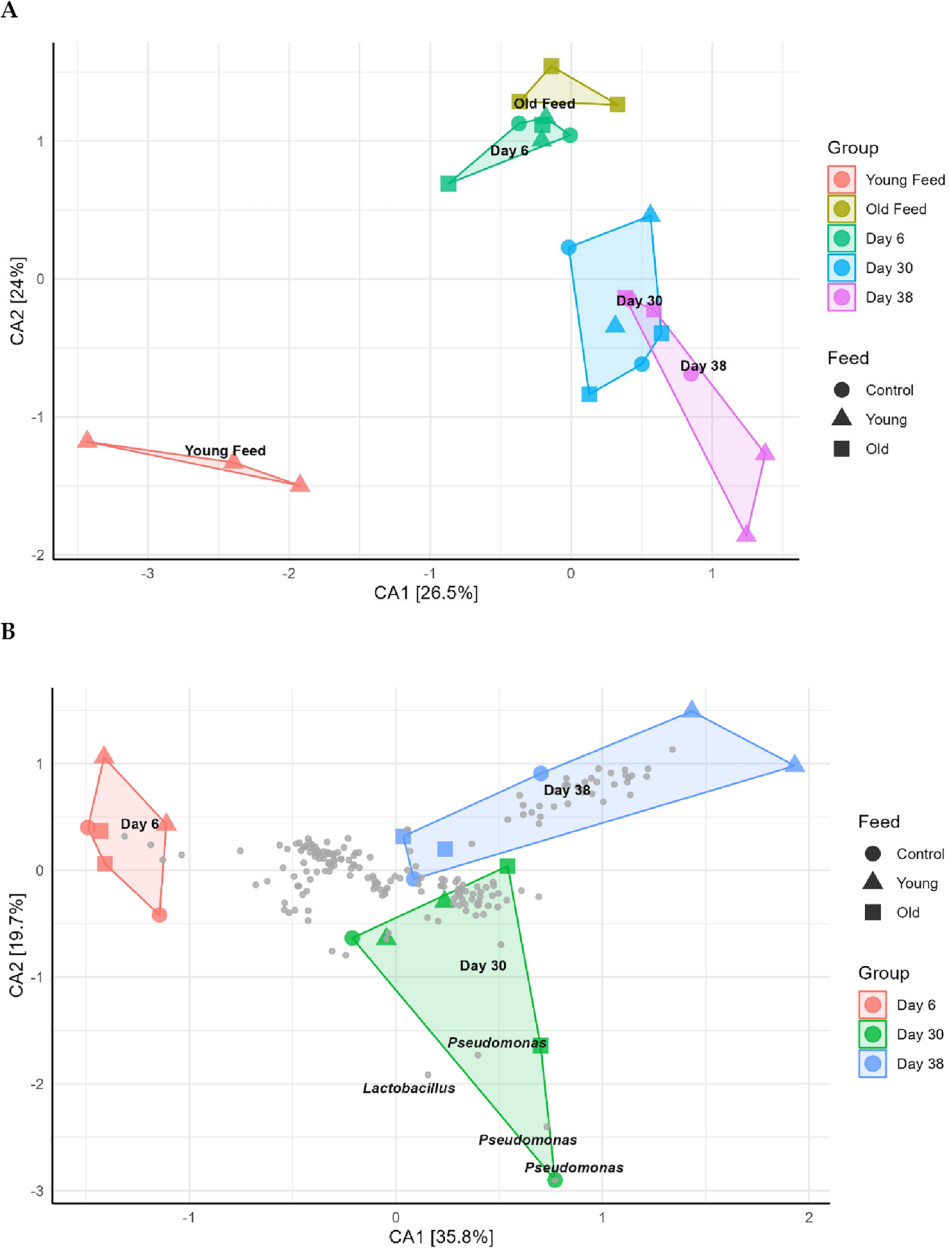
Correspondence analysis of microbiome samples and feed samples, based on Bray–Curtis distances. (**A**) CA plot of all microbiome samples in addition to Young and Old feed. (**B**) CA plot of microbiome samples with labelled species plots. For both figures, the age of the flies is distinguished by colour. This is also true for the different feed solutions in panel A. The type of feed is illustrated by differently shaped sample points.

The Correspondence Analysis (CA) revealed three clearly separated clusters. The Young feed clustered by itself away from the cluster with the Old feed and Day 6 fly microbiome samples, and the Day 30 and 38 fly microbiome samples constituted a third cluster (Fig. 2A). This mirrored the age dependent changes in the diversity of the microbiome (Table 1). The 38-day old flies fed the Young feed solution appeared to undergo the biggest change as they separated distinctly from the rest of the 38-day old flies. Age was found to be the most important factor explaining the differences between groups (ANOSIM, *p* < 0.001, R = 0.6281) as opposed to the feed which did not show an impact on the observed differences (*p* = 0.429, R = 0.0013) (Fig. 2A). For the 30 day old fly microbiome samples, the age dependent separation appeared to be characterized by a few genera in the samples, namely *Pseudomonas* and *Lactobacillus* (Fig. 2B). Most of the fly microbiome samples for day 30 and day 38 gravitated towards origo together with most of the observed OTUs, suggesting a potential shared composition.

### Microbial enrichment can modulate microbiome composition in aging flies

**Figure 3:**
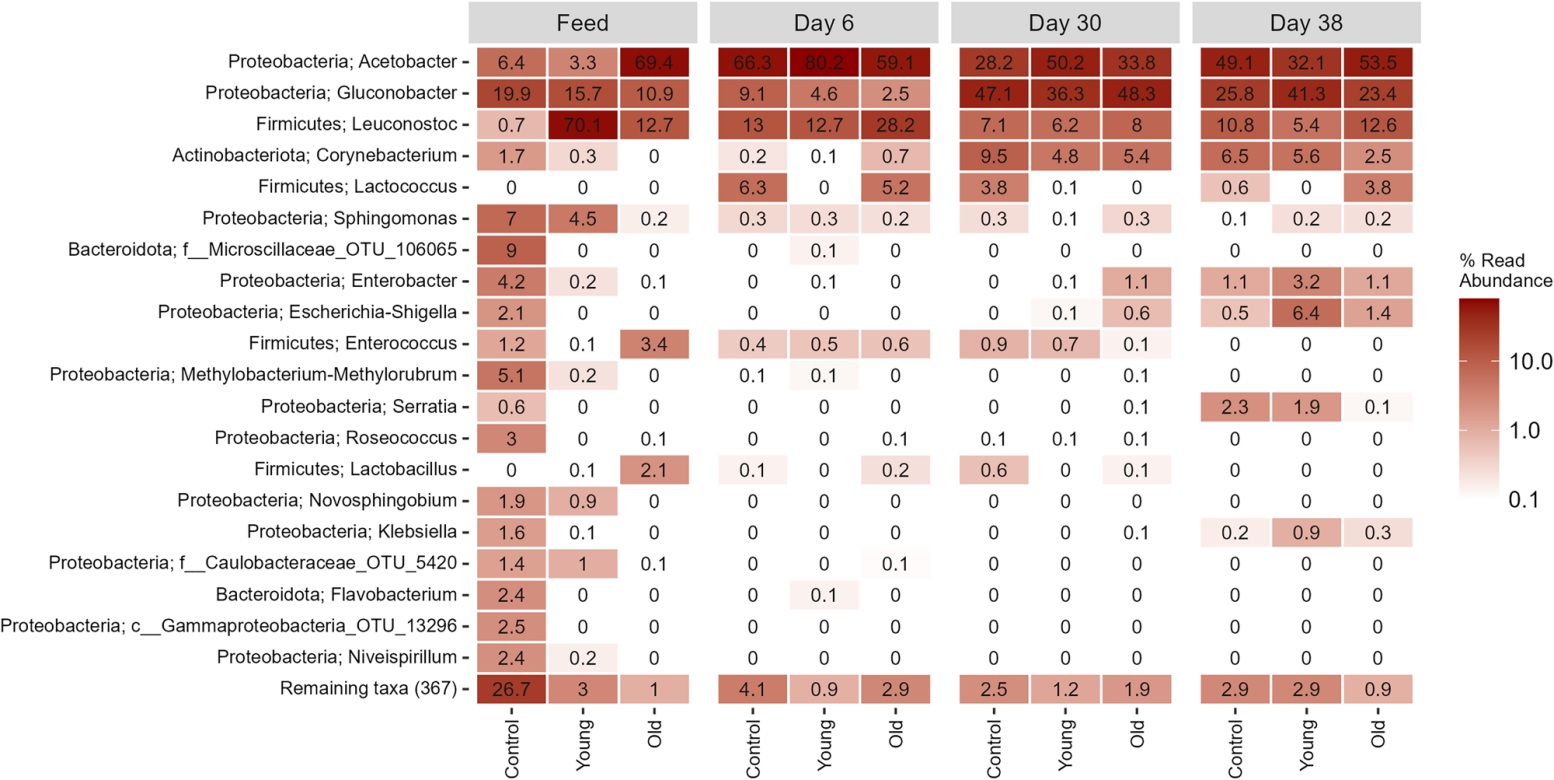
Heatmap of the 20 most abundant genera in addition to the relative abundance of the remaining taxa, across all samples, with the best possible taxonomic classification shown.

A heatmap revealed that the genera *Acetobacter, Gluconobacter* and *Leuconostoc* were the most abundant genera across both feed solutions and fly microbiome samples (Fig. 3). Additionally, it was observed that the microbiomes in the two different feed solutions differ, in that *Leuconostoc* was very abundant in Young feed while *Acetobacter* was the most abundant in the Old feed. Interestingly, *Lactobacillus* and *Enterococcus* which were abundant in the Old feed samples were not observed in the microbiomes of the older flies. Also, many of the abundant genera in old flies like *Escherichia-Shigella* and *Enterobacter* were not present in the Old feed solution. A similar situation was observed in the Young feed. Here *Leuconostoc* dominated, but this genus was much less abundant in the microbiome of the young flies. The age dependent changes in the microbiome composition (Fig. 2) were similarly observed in the heatmap where clear changes were seen between different days, with minor differences between the feed regimens (Fig. 3). The genera *Acetobacter* and *Leuconostoc* constituted the majority of the relative abundance in younger flies, but they experienced a decrease in relative abundance with increasing age of the flies. As the flies increased in age, so did the relative abundance of first *Gluconobacter* and *Corynebacterium* at day 30 followed by *Enterobacter, Escherichia-Shigella* and *Serratia* at day 38. This increase in relative abundance of certain genera occurred alongside the decline in relative abundance of the previously mentioned abundant genera resulting in a more even distribution of the present genera.

The genera *Enterobacter*, *Escherichia-Shigella*, and *Klebsiella* were only present in the 38-day old flies, and 30 days old flies who were fed the microbial feed solution from old flies. The opposite was observed for *Enterococcus* where we observed a drop in relative abundance with age and this genus is not present in the 38-day old flies and underrepresented in the 30-day old flies reared on the Old microbial suspension. For the 38 days old flies it was also observed that the relative abundance of the genera *Gluconobacter*, *Enterobacter*, *Escherichia-Shigella* and *Klebsiella* experienced an increased abundance in the flies reared on the Young feed solution as opposed to both the control and the Old feed solutions.

### Effects of enrichment of microbiomes using microbial supplementation on lifespan and negative geotaxis

**Figure 4:**
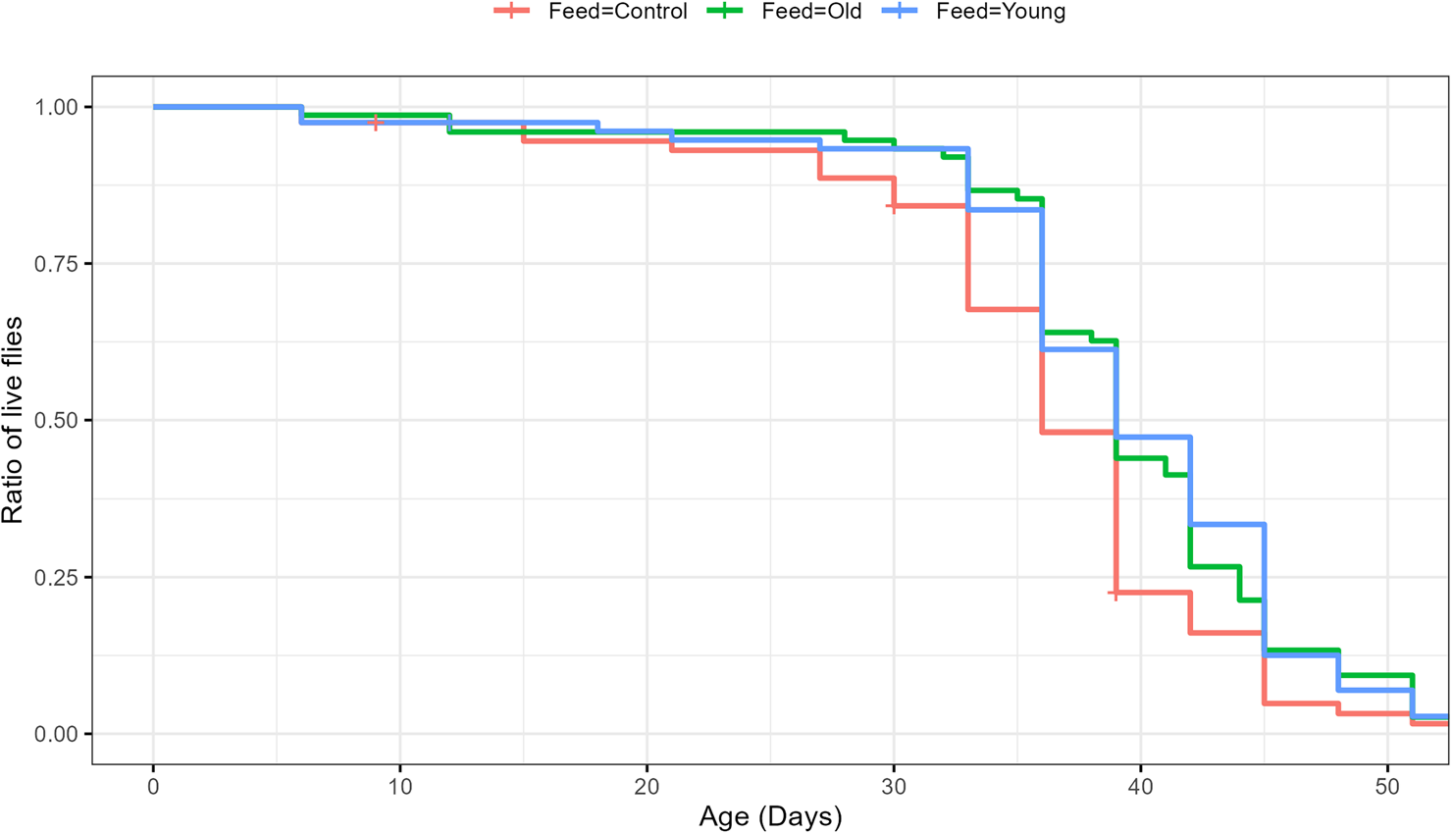
Microbial supplementation positively affects lifespan. Lifespan of D. melanogaster males is positively affected by supplementation with bacterial suspension from both old and young flies as compared to sucrose control (*p* = 0.034 and *p* = 0.0087, log-rank). No difference is observed between the two microbial supplements (*p* = 0.5, log-rank) (n = 80).

In parallel with the microbiome analysis, lifespan assessment and a RING assay were performed to quantify the potential health/fitness effects provided by the microbiome enrichment. The lifespan assay revealed an average lifespan of 39.2 days for the flies enriched with either the Young or Old gut suspensions while flies fed the Control diet only reached an average age of 35.8 days (Fig. 4).

All three feeding groups were stable, with only a few deaths until day 27 where the control group began to die at a higher rate. This drop was not observed in the groups supplied with bacterial feed solutions until day 33, almost a week later. All three groups then die off at a similar rate until day 54 which was the maximum lifespan for all three groups.

**Figure 5:**
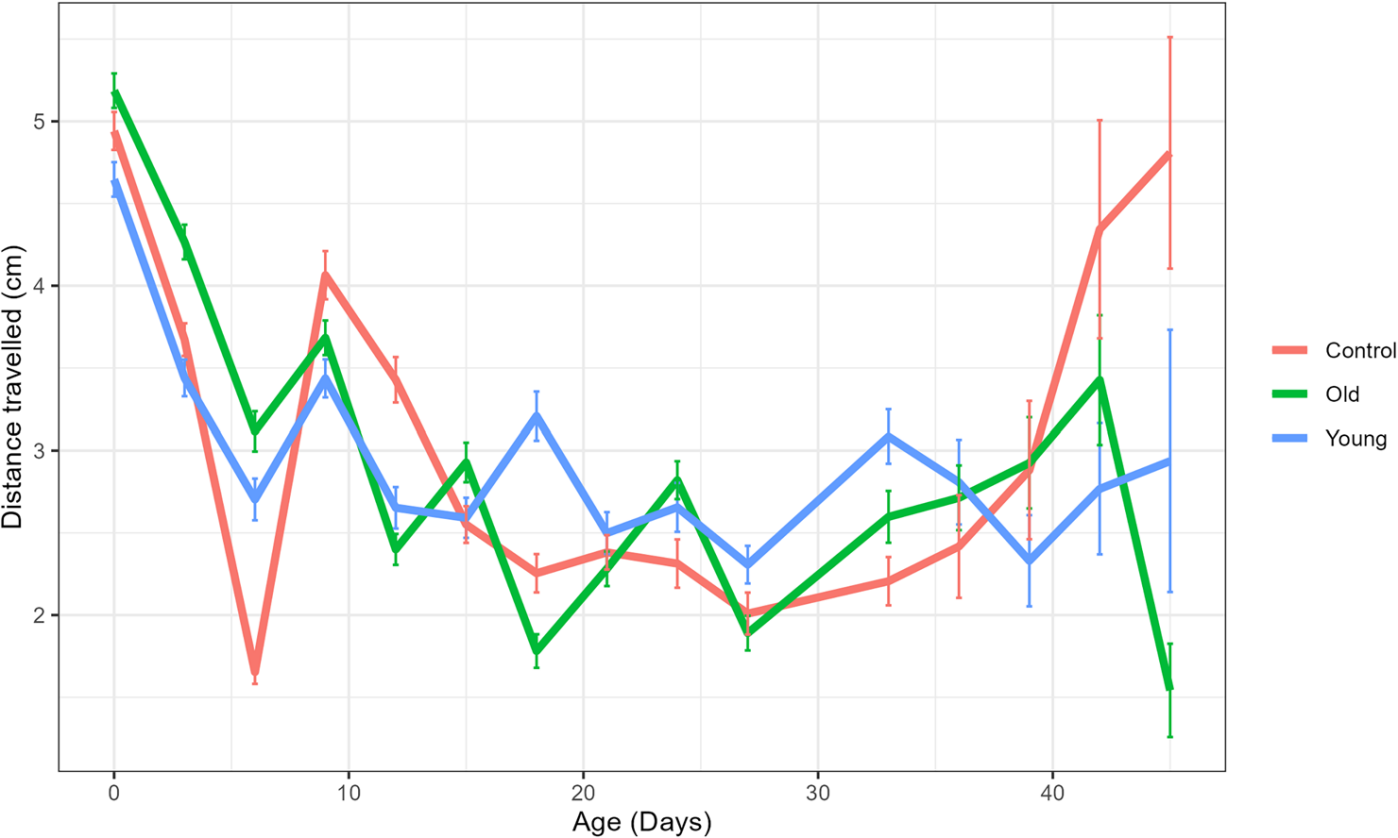
Negative geotaxis decreases with age regardless of microbial supplementation. Mean of means (± SE) for the length travelled (cm) in each tube as a measure of activity. A significant effect of age was observed (*p* = 0.036) and except for the Control feed, a decrease in negative geotaxis with increasing age was seen. The three feed types did not differ in negative geotaxis (*p* = 0.87), and the interaction between age and feed solution was also not significant (*p* = 0.26) (n = 130).

Additionally, a RING assay was performed in which the activity of flies was measured every 3 days to quantify the activity of the three groups across the lifespan of the flies (Fig. 5). A dip in activity around day 6 was observed across all three groups, followed by a decrease until day 27 after which all groups appeared to increase slightly in activity. Across the lifespan of the flies, a significant decrease in activity was observed as the flies aged, with the exception of an increase in the flies fed the Control solution at old age. However, no significant differences could be identified as a result of the supplementation of either microbial solution (Fig. 5).

## Discussion

The important relationship between the microbiome and multiple fitness components of the host has resulted in great interest in microbiomes in medical research, but also more broadly in biological sciences^9,14,15,59^. With the increased knowledge on interactions between microbiomes and host health, multiple therapeutic tools targeting this interaction have been developed, including probiotics and using fecal microbiota transplants^60,61^. Thus, to improve the efficacy and better understand potential uses, it is important to obtain a more nuanced understanding of the effects of microbial modifications on the host. Here we used the model species, *D. melanogaster*, to test if it was possible to shift the microbiota of aging flies by supplementing their diet with bacterial suspensions from the gut of differently aged flies. Our aim was to study microbiomes in *D. melanogaster* across age classes, and whether feeding flies with bacterial compositions harvested from young and old flies affected the microbiome compositions of recipient flies. We also investigated whether lifespan and behaviour of flies were impacted by feeding flies a Young or an Old microbiome. We observed that the microbiome differed markedly in gut content harvested from young and old flies, that microbiomes were different in flies fed bacteria from young and old flies, and that old flies generally had a distinct and, in contrast to expectations, more diverse microbiome compared to younger flies. However, we did not find evidence the feeding with either Young or Old gut content had an effect on the measured phenotypes. Hence, our working hypothesis that constitutively supplementing flies with microbes from young flies would manifest in increased life- and healthspan was rejected. While no effect was observed for the lifespan across the two microbial feed supplements, flies fed microbial suspensions (Old and Young) had increased lifespan when compared to those fed the control sucrose solution. This suggests that the age-related changes observed as result of the different enriched feeds have no effect on development and aging, but that compared to the control feed the enrichment conferred an advantage to the lifespan of the recipient flies. While previous studies have suggested that increased bacterial load after supplementation of aged microbiotas result in reduced fitness^46^, this method of utilizing non-sterile flies and thus using the supplementation to shape the microbiome rather than supplanting it provides interesting insights into the potential utilization of microbial supplements. However, we cannot rule out that increased lifespan of flies fed enriched diets is simply a consequence of increased nutritional content in these samples (e.g. from nutrients assimilated in the guts or from the structural components of the guts) and not being directly linked to the microbes. For the RING assay, no difference was observed between any of the feeding treatments except for an increase in activity of the flies fed the control solution after 40 days of age. However, considering that no significant effect was observed at earlier ages, and that less than 20% of flies were alive at this age, this increase is likely a result of the survival and test of the most fit flies, rather than an increase in motility representing the population as a whole.

We found that the genera *Acetobacter, Gluconobacter* and *Leuconostoc* were consistently observed amongst the most abundant in all samples which is consistent with the literature showing that these genera constitute members of the core *Drosophila* microbiome^45,62,63^. We also found that the relative abundance of these core genera (and others) differed markedly across life in the flies and that generally microbiome diversity increased with increasing age of the flies. This was also clear when comparing the Old and Young microbial feed suspensions, both regarding alpha and beta diversity, in that the composition of the two was notably different, where the Young suspension was dominated by *Leuconostoc* and the Old dominated by *Acetobacter*. In the human literature, the data is mixed regarding associations between gut microbial diversity and age. There is evidence for increased microbial diversity with increasing age in humans^64^, but also that healthy old people have a more diverse gut microbiome compared to similar aged but unhealthy old people^33^. This fits well with our findings (although we were not able to test it directly) where we investigate the healthy cohort of the old flies as the unhealthy flies were not available for investigation since they were dead prior to the collection time points.

In our experiment we fed flies with different microbial feed supplements, and a definite selection of microbial partners was observed in the flies. Though the flies were supplied with quite different microbial feeds, they were observed to have very similar microbial compositions between the three feed regimens. This microbial selection/curation of the host seem to be a conserved trait and similar results have been found in humans and other model organisms^41,42,65^. Despite this selection revealing quite similar microbiomes across the feeding regimes, certain important age-related differences were observed among the microbiomes of flies fed with different microbial supplements. Notably, the presence of *Enterococcus* in the samples appeared to be linked to younger *Drosophila* microbiomes, in that this genus was observed to be present in all microbiome samples from day 6, and in all microbiome samples from day 30 except for those reared on the bacterial supplement from the old flies. Likewise, the *Enterococcus* genus was found in much lower abundance in the 38 days old flies. The opposite trend was observed for the genera *Enterobacter, Escherichia-Shigella* and *Klebsiella*. These genera were observed to be represented in a much higher abundance in the microbiome of the 38 days old flies and the 30 days old flies which have been supplemented with microbial suspensions from old flies while being relatively underrepresented in the other samples.

*Enterococci* are a genus of hexose fermenting bacteria commonly found in nutrient rich resources, and it is unsurprising that it is found in the microbiomes of flies reared on sucrose solutions^66^. Certain species have previously been shown to stimulate the development of *Drosophila*^67^. However, considering that the relative abundance of this genera is diminished with age it appears there is selection against it, either due to competition with other microbes or from the host. This age-dependent selection favours the genera, *Enterobacter*, *Escherichia-Shigella* and *Klebsiella*, of which *Enterobacter* species have been linked to age related neurodegeneration in *Drosophila*^68^. This could suggest that aging favours an increase of *Enterobacter* which in turn negatively affects the *Drosophila* host which is in line with the theory of loss of immunocompetence as a driver of microbiome composition. This also supports the presence of *Shigella* which includes pathogenic species in *Drosophila*^69^. While the observed age-related changes in the microbial composition were not mirrored in the observed composition in the supplied feed, it supports the ability to alter the microbial composition using feed supplementation. Thus, it is possible to achieve a certain microbiome state via the use of microbial supplements. However, the unpredictable nature of the changes observed in this work, highlights the complex interactions and the need for further research.

In conclusion, we found evidence that microbiomes differ markedly across age-classes of *D. melanogaster*, and that by feeding flies suspensions with microbiomes of different ages we can alter the microbial composition of the recipient flies’ microbiomes. However, despite successfully altering the microbiome by feeding flies suspension with microbiomes from flies of different ages, we did not observe effects on the assessed phenotypes. When interpretating these results it is important to remember to be critical and not become a victim of the hyperbole associated with the importance of microbiomes for their hosts^70^. A simple explanation for the lack of phenotypic effects on the microbiome alterations performed in this study might be that our hypothesis was false and that life and health span of *D. melanogaster* cannot be improved using the presented procedure. However, more studies including both sexes, more populations and species, different diets and different feeding systems are needed to understand whether this conclusion is robust across conditions and genetic backgrounds across species.

### Supplementary Information

Supplementary Figures.

## Data Availability

All relevant sequence data can be found at the European Nucleotide Archive under accession number PRJEB71742.
