# Supplementary material for: Feeding Drosophila gut microbiomes from young and old flies modifies the microbiome
Source: Sci Rep. 2024 Apr 2;14:7799. doi: 10.1038/s41598-024-58500-1 (PMC10987527; doi:10.1038/s41598-024-58500-1)
Supplement: Supplementary file 1 — Supplementary Figures. [file 41598_2024_58500_MOESM1_ESM.pdf]

Feeding *Drosophila* gut microbiomes from young and old flies modifies the microbiome

Jonas Bruhn Wesseltoft, Christian Dupont Danielsen, Andreas Mølgaard Andersen, Nadieh de Jonge, Anders Olsen, Palle Duun Rohde and Torsten Nygaard Kristensen

Supplemental Material:

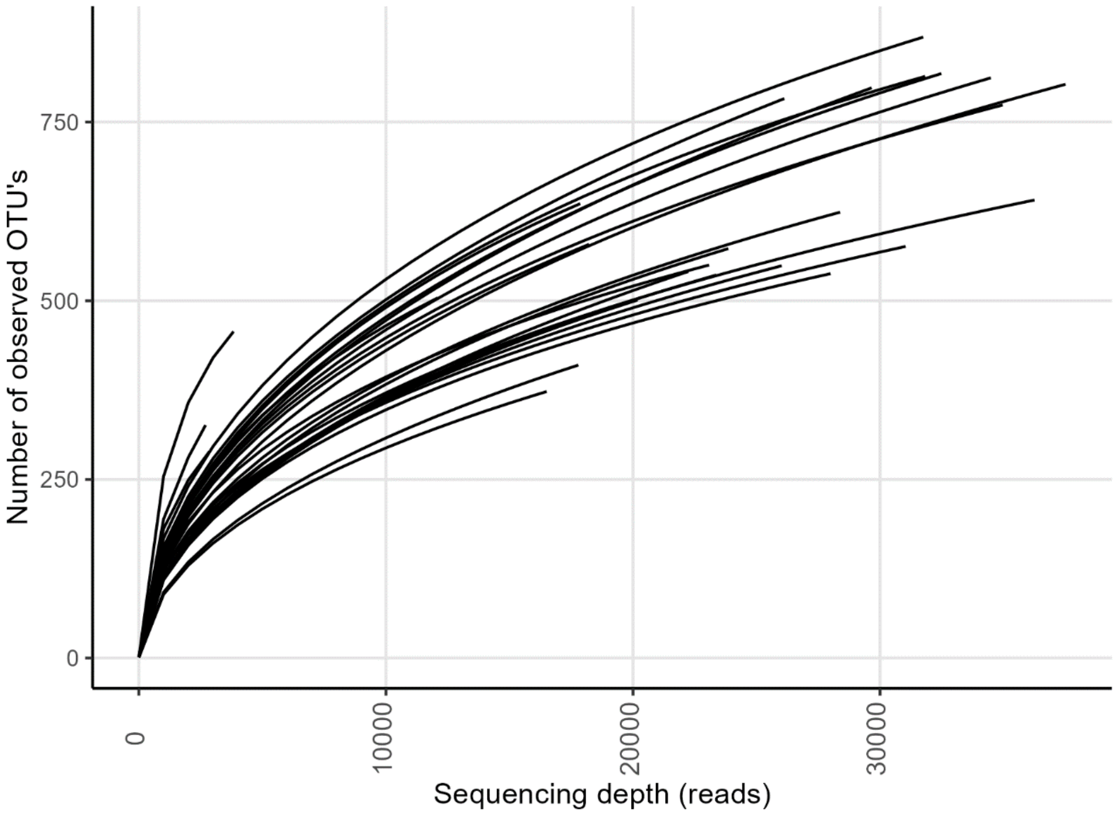

Figure S1: *Rarefaction curve of the individual samples.*

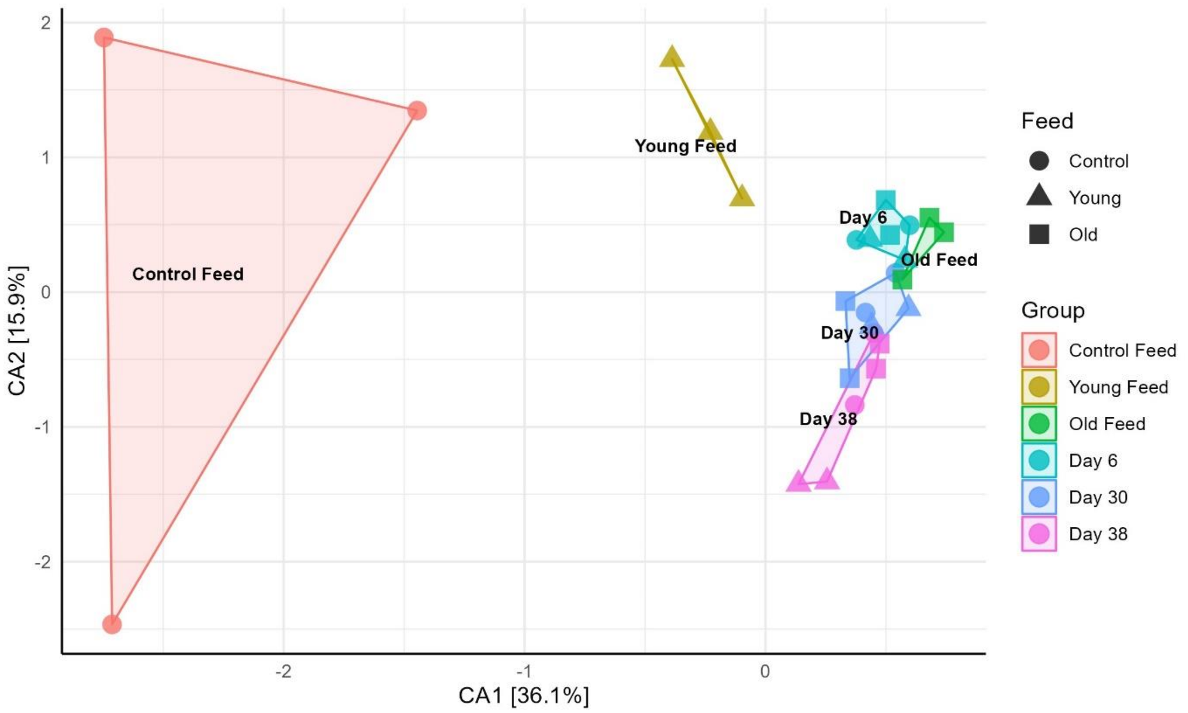

Figure S2: *Correspondence analysis of microbiome samples and feed samples, based on Bray-Curtis distances. The age of the flies is differentiated by colour. This is also true for the different feed solutions. The type of feed is noted by differently shaped sample points.*
